# Blood-Based Treatments for Severe Dry Eye Disease: The Need of a Consensus

**DOI:** 10.3390/jcm8091478

**Published:** 2019-09-17

**Authors:** Federico Bernabei, Matilde Roda, Marina Buzzi, Marco Pellegrini, Giuseppe Giannaccare, Piera Versura

**Affiliations:** 1 Ophthalmology Unit, Department of Experimental, Diagnostic and Specialty Medicine (DIMES), Alma Mater Studiorum University of Bologna, S.Orsola-Malpighi Teaching Hospital, 40138 Bologna, Italy; federico.bernabei89@gmail.com (F.B.); matilde.roda@hotmail.com (M.R.); marco.pellegrini@hotmail.it (M.P.); 2 Emilia Romagna Cord Blood Bank-Transfusion Service, S.Orsola-Malpighi Teaching Hospital, 40138 Bologna, Italy; marina.buzzi@aosp.bo.it; 3 Department of Ophthalmology, University Magna Graecia of Catanzaro, 88100 Catanzaro, Italy; giuseppe.giannaccare@unicz.it

**Keywords:** dry eye, ocular surface disease, autologous serum, allogenic serum, cord blood serum, platelet-derived eye drops

## Abstract

The use of blood-based eye drops as therapy for various diseases of the ocular surface has become increasingly popular in ophthalmic practice during recent years. The rationale for their use is based on the promotion of cellular proliferation and migration thanks to the supply of metabolically active substances, in particular growth factors. Blood-derived eye drops have been used for the treatment of several ocular surface disorders, such as dry eye disease, corneal ulcer, persistent epithelial defect, neurotrophic keratitis, ocular surface burn, recurrent corneal erosion, and limbal stem-cell deficiency. Both autologous (from patients themselves) and heterologous (from adult donors or from cord blood sampled at birth)-derived products exist, and each source has specific pros and cons. Despite an extensive literature, several issues are still under debate and the aim of this manuscript is to review the indications, preparation methods and storage, characterization of content, rationale for clinical outcomes, patient stratification, length of treatment, and rationale for repeated treatments at disease relapse. A rationale based on a “5 Ws and 2 Hs” protocol is proposed as a way of thinking, with the attempt to clarify Who, Why, When, Where, What, and How to use these treatment options.

## 1. Introduction

Dry Eye Disease (DED) is “a multifactorial disease of the ocular surface characterized by a loss of homeostasis of the tear film and accompanied by ocular symptoms, in which tear film instability and hyperosmolarity, ocular surface inflammation and damage, and neurosensory abnormalities play etiological roles” [1]. DED is common in the general population, increasing with age [2], and is one of the most frequent occurrences in daily practice [3], with a substantial impact on the individual’s quality of life [4]. Different modalities of treatment are available, with a proposed management algorithm based on a sequence of treatments according to the stage of disease [5]. The prescription of blood-derived products is recommended for the treatment of DED patients at stage 3 out of 4. 

The usefulness of treatment with eye drops prepared from autologous blood was first described in patients with chemical burns in the 1970s [6] and, ten years later, in patients affected by DED related to Sjögren’s syndrome [7], becoming popular thereafter and increasingly introduced in ophthalmic practice with other blood-derived products. These consist of eye drops derived either from patients’ own peripheral blood (autologous source) or from donors (allogeneic source—adult or umbilical cord blood) and prepared in the form of serum, platelet-rich plasma (PRP), plasma rich in growth factors (PRGF), and platelet lysate (PL) [8,9].

Blood-derived eye drops contain different biochemical constituents that act more closely to natural tears as compared to more conventional therapies [10,11], and the evidence of their effect on growth and proliferation of corneal epithelial cells has been investigated by in vitro and in vivo studies [12,13,14]. An increasing number of peer-reviewed papers have been published over the last fifteen years, showing the expanded indication for treatment, including mainly DED, persistent epithelial defect (PED), ocular graft-versus-host disease (oGVHD), recurrent corneal erosion (RCE), neurotrophic keratitis (NK), and limbal stem-cell deficiency (LSTD). Despite this extensive literature, there are still several issues under debate related both to the lack of a standardised protocol for preparation and storage and to the absence of consensus on the clinical strategy to achieve the best results, in particular, the rationale for clinical outcomes, patients’ stratification, length of treatment, and the rationale for repeated treatments. 

The aim of this manuscript is to review the indications, preparation methods, and clinical efficacy of blood-derived products used in ophthalmological practice, with a particular focus on unmet needs, current challenges, and future directions in clinical practice. 

## 2. The Rationale for Use of Blood-Derived Eye Drops

The “Ocular Surface System” includes the epithelia of cornea and conjunctiva, the limbus (junctional zone in between), the lachrymal and meibomian glands, the tear film, the eyelids, and the nasolacrimal duct. All components are functionally integrated and linked by the integrity of the corneal epithelium; by innervation; and by the endocrine, vascular, and immune systems [15]. 

The cornea is a transparent avascular tissue, accounting for approximately two-thirds of overall refractive power, with a metabolism strictly dependent upon diffusion of oxygen and nutrients from the tear film anteriorly and the aqueous humour posteriorly. The integrity of the corneal epithelium is enabled by self-renewing stem cells, located in the limbus at the Palisades of Vogt, which persists throughout its lifetime. The activity of corneal epithelial stem cells (CSCEs) is finely regulated by the surrounding niche. Moreover, different factors contained in the tear film are regulated by paracrine/autocrine pathways to allow maintenance of fundamental functions, such as cellular replacement and wound healing [16]. 

An increasing interest in the application of human-blood-derived products such as eye drops for DED arose over the last years. Serum is the most common blood product used as eye drops, which is obtained by the physiological clotting process of blood collected without an anticoagulant. The therapeutic benefits of blood-derived serum eye drops (SED) may be explained by a composition similar to that of tears [17,18,19]. SED contains carbohydrates, lipids, and various electrolytes like tears, a ten-fold higher total concentration of protein, and a mixture of epitheliotrophic growth factors [20,21] released by platelet alpha-granules degranulation that occurs during clotting [22].

Different types of growth factors (GFs) such as epidermal growth factor (EGF), fibroblast growth factor (FGF), transforming growth factor-beta (TGF-β), nerve growth factor (NGF), platelet-derived growth factor (PDGF), and insulin-like growth factor (IGF) and their receptors expressed in corneal epithelial cells, keratocytes, endothelial cells, and CSCEs stimulate turnover and proliferation of corneal cells and play a main role in maintaining ocular surface integrity [23,24]. To discuss the respective roles of these GFs is beyond the purpose of this manuscript, and readers are referred to reviews on this issue [25,26]. Finally, a reflection should be made on the continuous supply of such growth factors to the surface and, in particular, to the CSCEs which can undergo overstimulation, as it can be suggested by in vitro experiments. For instance, EGF increases proliferation of human corneal epithelial cells in a dose-dependent manner at concentrations greater than 0.1 ng/mL, but at concentrations exceeding 10 ng/mL, the cellular proliferation is reduced [23,27]. 

Therapeutic constituents of blood can be prepared by centrifugation, filtration, and freezing, all procedures which can influence the quality of the final product, also in terms of GF content. As the main focus is nowadays on using the specific component that is clinically indicated, processing procedures should be detailed and harmonized with an attempt to provide standardized blood products as the best of the existing options. For now, processing procedures are published and annually revised under the supervision of the European Directorate for the Quality of Medicines and HealthCare (EDQM; downloaded freely from https://www.edqm.eu/en/blood-guide) in the European Union. In the United States, blood and blood products are under the surveillance of the Food and Drug Administration (FDA; https://www.fda.gov/vaccines-blood-biologics/blood-blood-products). In Italy, according to the current legislation, recommendations for non-transfusional use of blood products is published by the Italian Society for Transfusion Medicine (SIMTI) [28]. 

## 3. Literature Review

### 3.1. Search Strategy

We searched PubMed, Embase, Web of Science, Ovid, Cochrane Database, and Scopus databases for original articles published up to 31 May 2019, using the keywords (all languages)(“Clinical trial*” OR “autologous serum*” OR “allogenic*” OR “cord blood*” OR “umbilical blood*” OR “PRP”) AND (“keratopathy” OR “dry eye” OR “neurotrophic keratitis” OR “corneal ulcer” OR “epithelial defect”) without any limitation. Moreover, reference lists of retrieved studies were manually scanned for all relevant additional studies and review articles. The searches were conducted by 3 independent investigators (F.B., M.P., and M.R.). Any discrepancies were resolved by discussion or by input from another reviewer (P.V.).

### 3.2. Study Selection

After removing duplicates, 2 authors (F.B. and P.V.) individually screened titles and abstracts of all identified citations. The full text of citations deemed as potentially eligible were obtained and individually screened for eligibility, and any disagreement was discussed with all authors. 

### 3.3. Eligibility Criteria

The articles were considered eligible if the studies met the following inclusion criteria: (1) study type: randomized controlled trial (RCT), retrospective; (2) population: patients having DED of any etiology, NK, PED, and RCE. Exclusion criteria were as follows: (1) lack of clinical data reporting; (2) types of publications other than original articles (e.g., abstracts from congresses, letters to editors, correspondence, reviews, duplicates, and full texts without raw data available for retrieval).

### 3.4. Data Extraction

Three reviewers (F.B., M.R., and M.P.) independently extracted the following data from each included publication: first author name, year of publication, design of the study, condition treated, inclusion criteria, number of patients and control subjects (if applicable), control arm product, solvent composition and dilution of the blood-based product, posology and duration of treatment, main outcomes, results, and retreatment parameters (rationale, cycles, time interval of treatment suspension, and total duration of treatment). 

Studies with data reported in figures or with missing data were excluded. In case of disagreement among the 2 reviewers, full texts were revisited and agreed on by discussion. 

### 3.5. Quality Assessment

All selected studies were graded according to the modified American Academy of Ophthalmology Preferred Practices guidelines [29] as follows. Level 1: Evidence obtained from at least one properly conducted, well-designed, randomized, controlled trial or evidence from well-designed studies applying rigorous statistical approaches. Level 2: Evidence obtained from one of the following: a well-designed controlled trial without randomization; a well-designed cohort or case-control analytic study, preferably from one or more center; or a well-designed study accessible to more rigorous statistical analysis. Level 3: Evidence obtained from one of the following: descriptive studies, case reports, reports of expert committees, and expert opinion. 

## 4. Autologous Serum Eye Drops 

Serum is the component remaining after clotting of whole blood, and it contains many of the factors present in tears. Autologous SED (auto-SED) is obtained from patients’ own peripheral blood serum. Production methods including clotting time, centrifugation parameters, dilution, storage time, and temperature were shown to determine quality and characteristics of the final dispensed product, but no consensus on technical details for the preparation of auto-SED have been established so far. Hence, the formulation and dilution factor of SED for DED treatment largely rely on the experience of single blood centers according to national or regional blood establishments, as was discovered in a recent survey conducted by the Biomedical Excellence for Safer Transfusion (BEST) Collaborative on methods used at international levels to prepare SED [30]. 

The several studies reviewed followed, with light differences, the basic protocol proposed by Liu [31] and others. Briefly, 50–100 mL of whole blood is taken from the patient and is left for 2 h at room temperature until complete clotting is reached. Then, the blood is centrifuged at 3000 g for 15 min to completely separate serum from solid constituents. Next, balanced salt solution (BSS) or isotonic saline solution is added to the supernatant until the desired concentration is reached. Allo-SED has to be stored at −20 °C for a maximum of 3 months, and it must be kept away from light to avoid vitamin A degradation. 

### 4.1. Pending Issues

#### 4.1.1. Dilution

The BEST survey reported that almost half of the centers dilute serum before dispensing in order to reduce to a physiologic concentration the levels of potentially anti-proliferative factors, such as TGF-β, which has shown to slow down corneal healing and to facilitate corneal stromal fibrosis and opacity in vitro [23,32]. Indeed, it has been shown that TGF-β plays a main role in the development of mature myofibroblasts during corneal healing after injury, producing a disorganized extracellular matrix in the stroma that results in loss of transparency [33]. Nevertheless, serum dilution leads to decreased levels of trophic factors, potentially limiting its beneficial effects. A recent Cochrane, which reviewed four clinical trials on auto-SED, reported the use of 20% dilution in all the studies [34]. Several studies investigated the use of higher concentrations (50 to 100%), reporting an excellent clinical efficacy and no adverse effect [35,36,37,38]. Moreover, other authors proposed to dilute SED to 20% in a sodium hyaluronate solution to improve retention time and to decrease the frequency of administration and reported good results. [39]. Given these evidences, it cannot be excluded that formulation may have to be adjusted to the treated disease or to its extent.

#### 4.1.2. Storage

Significant differences were reported in terms of recommended maximal storage time for serum drops, in part, due to internationally varying laws/regulations. It has been reported that several serum GFs are temperature and time resistant but substance P and calcitonin gene-related peptides significantly degrade at 4 °C within 24 h [40]. Hence, it is important to store serum vials frozen to preserve the activity of epitheliotrophic factors [41], which have been shown to be maintained until a prolonged storage of 6 months at −20 °C [42].

#### 4.1.3. Safety

Exposure to contaminated SED should be avoided, especially in DED patients who have a higher risk of developing infections because of their diseased surface. SED are prepared based on the guidelines for good manufacturing practice (GMP) [43], generally in open systems with a remote but still potential risk of contamination during the manufacturing process. Very few cases of adverse events related to contamination have been reported in the literature (reviewed in Reference [44]). To improve safety, sealed manufacturing systems (which package the eye drops into vials or long lengths of tubing, which are then heat-sealed to produce single-use devices) have been proposed in the market [45,46,47], but financial and logistic barriers are still unaffordable in many centers [48].

### 4.2. Clinical Results

Six RCTs investigated the efficacy of auto-SED for the treatment of severe DED (Table 1) [49,50,51,52,53,54]. Improvement of symptoms and signs (tear breakup time (TBUT), corneal staining, and conjunctival impression cytology) was reported in severe DED patients after therapy with auto-SED and tear substitutes with [50,51] or without [49] significant differences between the groups. In a cross-over study, severe DED patients were randomized to receive 3 months of treatment with auto-SED 50% and 3 months with conventional therapy, and viceversa; symptoms and impression cytology of conjunctival epithelial cells improved more significantly after treatment with auto-SED [50]. Two recent cross-over trials on severe DED reported a higher decrease of symptoms score in the auto-SED group compared to the control group treated with tear substitutes [52,53]; nevertheless, a significant improvement of TBUT was also reported in the auto-SED group only in the study from Celebi [53]. Yilmaz et al. reported a significant magnitude of the mean improvement of symptoms and TBUT in patients with DED due to systemic isotretinoin after auto-SED therapy compared to those treated with tear substitutes [54].

Several non-randomized studies evaluated the efficacy of auto-SED in DED of different aetiologies. Ogawa et al. investigated the use of auto-SED in patients affected by oGVHD, reporting a significant improvement of symptoms, TBUT, and corneal staining [55]. Two prospective studies evaluated the use of auto-SED in patients affected by primary and secondary Sjögren Syndrome (SS), with an improvement of both signs and symptoms only in patients with primary SS [56] or of only symptoms [57]. Both undiluted and diluted 20% auto-SED seem to ameliorate DED related to SS. In particular, Cho et al. [37] treated patients with SS DED (not distinguishing between primary and secondary), non-SS DED, and PED with undiluted auto-SED, 50% auto-SED in saline, or 50% auto-SED in sodium hyaluronate, reporting different results for different aetiologies. Jirsova et al. [58] treated DED associated with primary Sjögren, secondary Sjögren, sarcoidosis, and unknown aetiologies with 20% auto-SED in saline and reported significant improvements of clinical findings and symptoms occurring in 77% and 63% of eyes, respectively, but they did not stratify patients according to aetiology.

López-Garcia et al. [39] treated SS DED (not distinguishing between primary and secondary) with 20% auto-SED in saline or 20% auto-SED in sodium hyaluronate and compared the efficacy of two different dilution vehicles for auto-SED delivery. They demonstrated that both sodium hyaluronate and saline determine a significant improvement of tear-film stability, corneal staining, TBUT, and symptoms but without significant differences.

The potential efficacy of auto-SED treatment was investigated also in other ocular surface diseases, in particular NK, PED related to different causes (i.e., post refractive surgery, post perforating keratoplasty, and other ocular surgery), RCE, and LSCD (Limbal Stem Cell Deficiency) [59,60,61,62,63,64,65].

In a retrospective case series, auto-SED treatment for NK refractory to conventional therapy lead to complete healing in 100% of patients and to an improvement of corneal sensitivity in 64% of patients [59]. A large prospective study evaluated undiluted auto-SED treatment for PED due to different types of ocular surgery and reported a complete corneal healing in more than 90% of patients on an average of 4 days [63]. In a case-controlled study on pterygium surgery, auto-SED promoted a faster corneal re-epithelialization as compared to tear substitutes [64].

A randomized trial compared auto-SED treatment with tear substitutes after laser in situ keratomileusis (LASIK) and reported an improvement in TBUT and rose bengal staining in the auto-SED group, with no difference in symptom scores between groups [60].

In a large case-controlled study that compared auto-SED with tear substitutes after PK, patients who received auto-SED showed a significant improvement of corneal re-epithelialization time. Moreover, the efficacy of auto-SED was reported even in difficult cases such as larger-diameter grafts and diabetic patients [65].

Schulze et al. compared autologous serum therapy with hyaluronic tears in diabetic patients undergoing pars plana vitrectomy who received corneal abrasion for better intraoperative visualization [61]. Patients treated with auto-SED showed a significant improvement of the closure of corneal epithelium compared to the control group [61].

A prospective study investigated auto-SED treatment in patient RCE refractory to standard therapy. After auto-SED treatment, complete corneal healing was reported in 100% of cases, and in 85% of cases, no relapse occurred during the following 12 months. [62]. Auto-SED treatment in 13 patients affected by LSCD due to aniridia lead to an improvement of tear stability, corneal epithelialization, and symptoms [66].

Although the encouraging results reported in the RCTs on the efficacy of auto-SED therapy for severe DED, a Cochrane meta-analysis published in 2013 and based on 4 auto-SED RCTs showed an overall inconsistency in patient’s reported symptoms and lack of effect based on objective clinical measures, concluding that high-quality RCTs are warranted to assess the benefits of this treatment in DED [34]. The 21 studies on auto-SED included in the present review had levels of evidence 1 (9 studies) or 2 (11 studies) and only one study had level 3, with only 11 studies analyzing auto-SED with a comparator, generally unspecified tear substitutes. In agreement with the Cochrane report, however, the inconsistency of measures and standardized preparation prevent a conclusive statement on auto-SED efficacy as compared to conventional treatments.

## 5. Allogeneic Adult Peripheral Blood Serum Eye Drops

The rationale for the use of SED prepared from donor sources is now driven by the assumption of negative effects caused by serum-derived autoantibodies or pro-inflammatory cytokines that would be deliberately applied to the ocular surface in patients with important comorbidities. Peripheral blood from adult donors and cord blood sampled from umbilical veins at birth are now eligible candidates for allogeneic sources. Although SED of autologous origin is still the most manufactured product, an increasing number of centers worldwide is now producing SED from allogeneic blood donors [67,68,69,70,71,72,73,74,75]. When the SED are from allogeneic origins, procedures must be undertaken to match all blood groups to ensure hemato-immunological ABO antigen matching between donors and recipients. One option could be to prepare SED from AB group donors to hold a single blood group source, but the limited prevalence of the AB group in the Caucasian population may limit the availability of this source.

The use of allogenic serum obtained from healthy donors has been introduced as a possible alternative to auto-SED in patients with inaccessible peripheral venous access or use of anticoagulant medications or coexisting systemic diseases such as coagulation factor deficiency and haematological disease. Moreover, some authors reported that peripheral serum of SS and oGVHD patients that represent two of the most common causes of severe DED might contain high concentrations of pro-inflammatory mediators potentially harmful to the ocular surface [76], although others disagree [45]. In addition, it has been shown that other diseases are associated with an altered serum composition that presents significantly decreased epitheliotrophic properties, in particular, in patients with chronic renal failure and rheumatoid arthritis [77,78]. Other disadvantages of auto-SED are that some people fear venipuncture and the prolonged treatment, and the frequent drawing of blood can be inconvenient in others. Cultural considerations also play some role as, in Asian cultures, the belief is held that frequent blood sampling increases weakness and susceptibility to infections [69].

Preparation methods of allogeneic SED (allo-SED) are the same as that of auto-SED. The whole blood is taken and stored at room temperature until it is clotted. Next, serum is separated by centrifugation at 3000 g for 15 min, filtered, and diluted to 20% concentration [36]. Allogeneic blood donors donating blood for the production of SED should be screened for virus markers using the same procedures that are applied to donations used for transfused blood products [30,43,79].

The use of allo-SED also poses some issues related to legal or ethical concerns [80], and a written informed consent is required in many legislations.

### Clinical Results

Table 2 reports the list and details of the main studies related to the use of allo-SED.

Chiang et al. [69] investigated the use of allo-SED in patients affected by PED due to different causes, including penetrating keratoplasty (PK), NK, oGVHD, and exposure keratopathy, that were refractory to standard treatment. Relatives donated blood for the preparation of allo-SED that was used at a concentration of 100%. Almost half of the treated patients showed complete healing within 2 weeks that was reached in 63.9% of patients within 4 weeks.

Allo-SED efficacy was evaluated also in patients with DED related to oGVHD. In particular, after four weeks of treatment with 20% allo-SED obtained from healthy donors, a significant decrease in symptoms score, tear osmolarity, corneal staining, and TBUT was noted [72]. Furthermore, another study reported a significant improvement in symptoms and ocular surface parameters in 2 oGVHD patients treated with allo-SED [70].

Harritshøj et al. evaluated the efficacy of allo-SED 20% prepared from blood of identical ABO groups in patients affected by both DED and PED [71]. They showed a significant improvement in symptoms and signs in the DED group, while no significant changes were the PED group.

A randomized, double-blind study on DED patients evaluated comparatively by confocal microscopy the effect on the corneal sub-basal nerve plexus of a one-month treatment with two allogeneic products, namely peripheral blood serum from adult donor or umbilical cord blood serum (UCBS) eye drops [81]. Overall, both treatments significantly improved corneal sub-basal nerve plexus parameters, with a superiority for UCBS drops associated with a higher increase of the corneal nerve fractal dimension global metric.

Since 2007, 39% of all SEDs issued in New Zealand have been allogeneic [68], and a cross-over retrospective study was recently reported [74], dealing with a comparison of autologous and allo-SED in patients who served as their own controls. Results demonstrated a comparable efficacy and tolerability in both products, and most of those who changed from autologous to allo-SED reported either maintained benefits or further improvement, but a prospective randomized trial is now needed to confirm this observation.

## 6. Allogeneic Umbilical Cord Blood Serum Eye Drops

Umbilical cord blood serum is collected from mothers during both vaginal and caesarean delivery. An informed consent must be signed prior to performing the blood collection. From 10 to 20 mL of the umbilical cord blood is collected from the umbilical vein after delivery. The blood is clotted for 2 h at room temperature; after centrifugation at 3000 rpm for 15 min, the serum is isolated carefully under sterile conditions in a laminar air flow hood. Then, 20% concentration is reached by the dilution with BSS. The serum is aliquoted into sterile 1-mL vials with ultraviolet light protection. Sealed eye drop vials are stored in a freezer at −20 °C for a maximum of 3 months, and opened bottles are kept in a refrigerator at 4 °C [46]. To avoid the transmission of blood-borne diseases, tests for HCV, HBV, HIV, syphilis, toxoplasma, and CMV have to be performed.

As for allo-SED, UCBS represents a possible therapy for patients affected by systemic disease or other conditions that may contraindicate the use of auto-SED due to the presence of inflammatory mediators in the serum of the same. UCBS has a higher concentration of GF, such as EGF, TGF-β, NGF, and VEGF, compared to other blood derived preparations [46,82,83,84]. Nevertheless, UCBS contains a lower level of IGF-1 and vitamin A compared to peripheral blood serum but higher compared to tears [83]. It has been shown that blood samples collected from young mother (<30 years) has a higher concentration of EGF, along with longer labour duration (>6 h), and higher CD34+ cell content (0.05 × 10^6^/mL). This pre-selection may be helpful to obtain UCBS with the ideal concentrations in EGF for healing corneal wounds without the need of GF laboratory dosages [85].

### Clinical Results

UCBS eye drops were investigated as possible therapy of DED [46,82,86], PED [87,88,89,90], acute chemical burns [91,92], oGVHD [93], RCE [94], after PK [95], laser epithelial keratomileusis (LASEK) [96], Hansen’s disease [97], and NK [98,99] (Table 3). A significant reduction of subjective discomfort symptoms associated with an improvement of tear stability, and recovery of the corneal sensitivity and keratopathy after UCBS treatment was shown in all studies in severe DED patients, with a high degree of satisfaction upon instillation. PED refractory to previous medical management [87,88,89] showed faster healing, no recurrence three months after the end of UCBS treatment, and higher increase of goblet cell density as compared to autologous serum treatment in SS patients [90]. UCBS also was shown to significantly ameliorate sub-basal corneal nerve morphology by an in vivo confocal microscopy study, suggesting a role of neurotrophic factors contained in UCBS [84] in its mechanism of action. These positive results were confirmed also in severe DED associated with oGVHD, in which symptom score, corneal sensitivity, TBUT, and keratoepitheliopathy score improved significantly after 2 months of UCBS treatment, and the improvements were maintained by 6 months after treatment [93]. The treatment with UCBS was compared to auto-SED [91] and amniotic membrane [92] treatments in patients with ocular chemical burns, showing a higher effectiveness in the ocular surface restoration and, in particular, in complete re-epithelialization already after 3 ± 4 weeks and reduced corneal haze. Yoon et al. evaluated the efficacy of UCBS in patients affected by RCE, reporting a mean healing time of 4 weeks and a reduced number of recurrences [94]. UCBS seems to be useful in the treatment of NK as it contains high levels of neurotrophic factors such as substance P, IGF-1, and NGF. In particular, a study on 28 eyes with NK reported complete epithelial defect healing in all eyes, with a mean healing time of 4.4 ± 4.0 weeks [98]. UCBS was also used in adjunct to conventional treatments after LASEK and was successful in reducing the early postoperative corneal haze and in improving tear film and ocular surface parameters [96]. Both auto-SED and UCBS treatments were effective in restoring the tear proteomic profile and conjunctival cytology in DED associated with leprosy [97], which includes corneal anaesthesia and severe epithelial squamous metaplasia. The UCBS treatment was proven in maintaining longer these beneficial effects after treatment.

## 7. Platelet Derived Eye Drops

Platelets play a main role in the wound healing process, since they have high concentrations of GFs and cytokines contained in their α-granules, such as PDGF, TGF-β, and platelet factor IV [100].

Platelets-based preparations are extremely versatile; exist under forms of variable solidity from liquid to gel; and have been largely used in regenerative medicine, orthopaedic and maxillo-facial surgery in order to promote tissue healing through the delivery of several bioactive factors [22]. Various preparations (namely PRP, PRGF, and platelet lysate) have quite recently been introduced as possible therapy of different ocular surface disorders.

In platelet-derived eye drops, the autologous source still represents the most used product in DED patients, whereas allogeneic PRP, developed for other fields of application, is still at its beginning as an eye-drop treatment.

### 7.1. Preparations

There is a large heterogeneity among PRP separation systems regarding concentrations of platelets, leukocytes, and growth factors in PRP. The choice for the most appropriate type of PRP should be based on the specific clinical field of application. For each preparation, we detail below the most used in the treatment of DED, as they appear in the literature.

Platelet-rich plasma is obtained from plasma that has a platelet concentration of over 1 × 10^6^/mL. Thirty mL of the patient’s venous blood is drawn in tubes with 3.2% sodium citrate in order to prevent platelet activation prior to its use. A first centrifugation with low forces (10 min at soft spin from 200 *g* to 600 *g*) separates the whole blood into three layers: an upper layer that contains mostly platelets and white blood cells (WBC) called platelet-poor plasma (PPP); an intermediate thin layer of whitish color called buffy coat (BC), rich in WBC; and a bottom layer that consists mostly of red blood cells (RBC). The upper layer and superficial buffy coat are transferred into another sterile tube for a second centrifugation step at higher speeds (10 min at hard spin from 700 *g* to 2300 *g*) to concentrate platelets. The upper two-thirds of the volume (PPP) is discarded, while the lower one-third (5 mL of plasma) is homogenized by gently shaking the tube to create PRP. The product can be used either undiluted or diluted in BSS, sealed in vials or bottles, stored in the refrigerator at 4 °C for a maximum of 1 week or at −20 °C for longer periods. PRP acts by stimulating the release of PDGF, that is the first growth factor involved in the wound healing, and of TGF. Platelet-derived growth factor determines the increase of activated macrophages along with the development of new blood vessels. Transforming growth factor is able to induce chemotaxis and to control the epithelial proliferation, maintaining cells in an indifferent state. Furthermore, other important factors are the EGF that accelerates corneal epithelial proliferation and vascular endothelial growth factor (VEGF) and FGF-2 that take part in the angiogenesis process [101].

Plasma rich in growth factors. In the literature, a specific procedure is found, patented to standardize the PRGF preparation. Collected blood is centrifuged at 580 *g* for 8 min at room temperature in an Endoret System centrifuge (BTI Biotechnology Institute, S.L., Minano, Alava, Spain). The whole column of PRGF is collected after centrifugation using the Endoret ophthalmology kit (BTI Biotechnology Institute), avoiding the buffy coat that contains the leukocytes. The PRGF eye drops are incubated at 37 °C for 1 h; the obtained supernatant is then heat-treated at 56 °C for 60 min to eliminate the complement fractions and other immunologic components and to obtain the so-called “immunosafe PRGF”. Finally, plasma supernatants are filtered, aliquoted, and stored at −20 °C. Before starting treatment, patients are taught to preserve the PRGF eye drops at −20 °C for maximum 3 months and to use each dispenser for 3 consecutive days [102].

Platelet lysate—Sixty mL of anticoagulated peripheral blood is collected from each patient and centrifuged to obtain an autologous platelet concentrate. The concentrate (at 0.7 × 10^6^/mL) is frozen and thawed (thermal shock −80 °C/+37 °C) to lyse platelets and GF release. The lysate is then diluted with sterile, balanced saline solution (30% *v/v*) and aliquoted as 30 ready-to-use, sterile doses (ColSystem, Biomed Device Modena, Italy). A sample for microbiological investigations is taken at the time of preparation. Next, the final preparation is frozen at −20 °C and stored in the patients’ own freezer for a maximum of 45 days [103,104].

### 7.2. Clinical Results

Table 4 summarizes the characteristics and the main outcomes of clinical studies on the use of platelet-derived products for the treatment of ocular surface diseases. Fluorescein staining improved significantly in 72% of patients. The efficacy of PRP for the treatment of DED was investigated by Alio et al. who reported an improvement of symptoms in 89% of patients and a gain of at least 1 line of best-corrected visual acuity in 28% of cases. Furthermore, 56% of patients had improvements in the tear meniscus height from low to normal level; 86% of patients showed a significant improvement of conjunctival hyperaemia in slit-lamp appearance; and finally, fluorescein staining improved significantly in 72% of the cases [105]. The same research group treated with PRP 26 eyes suffering from post-LASIK DED, with improvements in symptoms in 85% of patients and a gain of 1–2 lines of best-corrected visual acuity in 54% of them [106]. Alio et al. also employed topical PRP eye drops in 26 eyes with chronic non-healing corneal ulcers and reported healing of the ulcer in half of the eyes and improvement in inflammation and subjective symptoms in all of them [107].

Lopez-Plandolit used autologous plasma rich in PDGFs in 20 eyes affected by PED unresponsive to conventional treatment, reporting healing of the epithelial defects in 85% of cases after a mean therapeutic time of 10.9 weeks [108]. In another study, patients with ocular oGVHD unresponsive to conventional therapy were treated with autologous plasma rich in PDGF eye drops. Photophobia and TBUT improved in 82.6% and 86.9% of patients, respectively [109]. In 2017, the same authors evaluated the long-term efficacy of plasma rich in PDGF eye drops in 31 patients with oGVHD. After 36 months, symptoms and TBUT significantly improved and no adverse events occurred [103]. Kim et al. compared the healing efficacy of allo-SED and PRP in 28 eyes affected by PED and reported a higher healing rate in the group treated with PRP [110]. A randomized controlled trial on 20 eyes affected by grade III to V chemical injuries compared PRP eye drops with standard medical treatment or standard medical treatment alone. After 3 months of treatment, significantly higher improvements in corneal transparency and in visual acuity were found in patients who received PRP [111].

In an in vivo confocal microscopy study, Fea et al. reported a significant increase in basal epithelium cells density and sub-basal nerve plexus density and a decrease in Langerhans cell density in patients with SS-related DED treated with platelet lysate [112]. Lee et al. compared the efficacy of PRP eye drops and tear substitutes in patients with RCE and found a significantly lower frequency of recurrence in eyes receiving PRP eye drops [113].

Merayo-Lloves et al. evaluated the safety and efficacy of autologous plasma rich in PDGF eye drops in 83 patients with evaporative DED and reported a significant improvement in symptoms, best-corrected visual acuity, and Schirmer test [114]. The same research group investigated the use of PDGFs in patients with SS-related DED [102], NK [115], post-LASIK DED [116], and oGVHD [117]. Patients with SS-related DED showed a significant improvement in symptoms and best-corrected visual acuity after treatment [102]. Patients with DED following LASIK surgery showed an improvement in symptoms and Schirmer test after treatment [116]. Patients with oGVHD showed an improvement of symptoms, corneal staining, best-corrected visual acuity, TBUT, and Schirmer test [117].

A complete resolution of corneal ulcer was reported in 97.4% of patients with NK after a mean time of 11.4 weeks [115]. Similar results were obtained in 25 NK patients treated by Wróbel-Dudzinska et al. with PRP eye drops, with complete healing of the corneal ulcer observed in 80% of patients, while improved symptoms and visual acuity observed in all of them [118]. In a randomized clinical trial, Garcia-Conca et al. compared PRP eye drops with tear substitutes in patients with hyposecretory DED [119]. Patients treated with PRP showed a significantly higher improvement in symptoms, visual acuity, conjunctival hyperaemia, corneal and conjunctival staining, Schirmer test, and tear osmolarity [119].

Due to its versatile methods of preparation, PRP can be used also as a solid preparation during reconstructive surgery of the ocular surface. A solid clot of PRP was used in combination with other surgical techniques, such as amniotic membrane transplantation and bovine pericardium membrane (Tutopach), to treat corneal ulcers and corneal perforations [120]. In a randomized controlled clinical trial, Avila et al. evaluated the administration of PRP as an injectable solution into the lacrimal gland in patients with SS-related DED. Compared to the control group, the intervention group showed a higher improvement in corneal staining, Schirmer test, TBUT, and symptoms [121].

## 8. Data Analysis and Criticism

Regulations vary from country to country for the use of blood-derived eye drops, which are classified differently according to local legislations—ranging from an unlicensed medicinal product (“special”) to “simple” blood component—with variable degrees of restrictions.

Clinical recommendations on SED for severe ocular surface disease have been published by the Royal College of Ophthalmologists [122,123], with the proposal to regulate SED by enrolling patients in a national program of outcome reporting that includes frequency and duration of treatment, serious adverse events or reactions, and patient self-reported outcomes. In this report, a 50% dilution in 0.9% sodium chloride is recommended (as provided by National Health Service Blood and Transplant, the only accredited SED production facility in the UK), only based on good practice points upon consensual expert opinion.

Data from our work were extracted from 55 papers of level of evidence 1 (*n* = 17) and 2 (*n* = 32), and only 6 papers were graded as level 3. All blood preparations used so far have been included for all sources and preparations of blood based treatments for ocular surface disease: auto-SED (*n* = 20, Table 1); adult allo-SED (*n* = 3, Table 2); allogeneic UCBS eye drops (*n* = 15, Table 3); allogeneic platelet products (*n* = 17, Table 4) subdivided into PRP (*n* = 9); platelet lysate (*n* = 3); and PRGF (*n* = 5).

All studies showed a good profile in terms of safety and efficacy for all the products.

**Figure 1:**
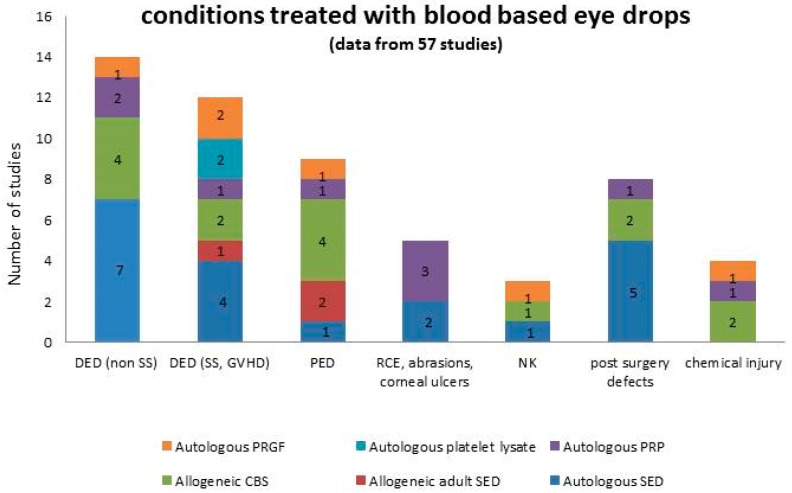
Summary of the conditions treated with blood-based eye drops, graphed in columns where each colour represents a specific product. Numbers in the columns represent the number of studies.

The list of ocular surface diseases treated with blood-based eye drops spans several conditions of different aetiology and is summarized in Figure 1. We were not able to find any information about the rationale for the product selection based on a specific condition or graded according to severity.

Data highlights the elevated heterogeneity of processing of blood-based eye drops despite the assumption that any procedure in the preparation may impact the content of the active substances or may influence their delivery. Sterile saline was the most used diluent, and it was reported in 43 out of 55 studies. The alternative use of sodium hyaluronate or carboxymethyl cellulose or unspecified tear substitutes were reported in 8 out of 55 studies with the aim to improve the retention time of the product or to increase its availability, but results were too few and inconsistent to provide comparable data.

Dilution 20% was the most used percentage, reported in 31 out of 55 studies, followed by 50% (7 studies) and undiluted (3 studies). The rationale for choosing a dilution was not discussed in the totality of the studies; in the PRP section, the dilution was not even reported in 14 out of 18 studies included in the analysis.

Taken altogether, data suggest that the parameters of processing and use were considered more as an established habit of the centers over the years rather than a parameter to be tailored.

The conditions treated with blood-based eye drops are summarized in Table 1. Moderate and severe DED, eventually associated to autoimmune diseases and oGVHD, and PED were the most represented diseases treated with all the products. No indication on the choice of a specific product for a specific disease was recorded in the analysis.

**Figure 2:**
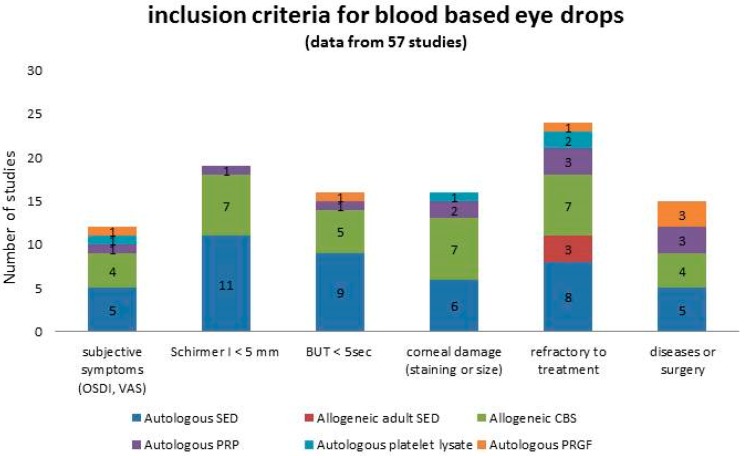
Summary of the inclusion criteria considered in patients entering a therapeutic program with blood-based eye drops, graphed in columns where each colour represents a specific product. Numbers in the columns represent the number of studies.

Inclusion criteria considered for a given patient to enter a therapeutic program with blood-based eye drops are summarized in Figure 2. To be refractory to previous treatment (ranging from tear substitutes to therapeutic soft contact lenses, topical steroids, and 0.05% cyclosporine) was the most represented indication for the prescription, followed by severely impaired parameters of tear functions and subjective symptoms of discomfort indicating severe dryness. Despite an increasing interest in other disciplines concerning the application of blood-based products to pursue reduction of pain [124], no indication of the prescription of blood-based eye drops focused on ocular pain relief was found.

Posology of treatment was also extremely variable, and when specified, it ranged from one drop every hour to 3, 4, 6, 8, 10, or 12 drops/day or to scaled regimens consisting of a higher frequency the first weeks of treatment and then decreasing. Beyond the obvious consideration that the amount of active compounds supplied relates to the posology, it is also an issue that tear substitutes may be necessary in adjunct to protect the surface in case of a regimen with few drops. No indication on eventual concurrent or rescue treatment allowed in cases of adverse events was found. No indication on dose size was found.

One month was the most reported time of treatment (8 studies), followed by 6 weeks (6 studies), 2 months (2 studies), 3 and 6 months (5 studies each), continuously for one year and until healing (2 studies each), or unspecified (in the remaining studies). No consideration on the eventual early stop of the treatment or on the retreatment parameters in terms of rationale to retreat and time interval of treatment suspension was found. The recurrence rate was considered in only two studies [62,94].

**Figure 3:**
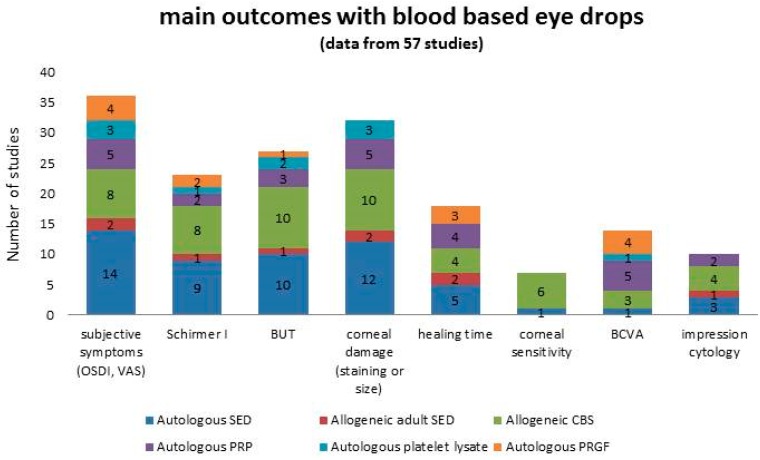
Summary of the main outcomes reported in patients treated with blood-based eye drops, graphed in columns where each colour represents a specific product. Numbers in the columns represent the number of studies.

Parameters considered as main outcomes are summarized in Figure 3. Reduction of subjective symptoms and reduction of corneal damage (expressed either as reduction of staining and/or area size) were reported in almost all the studies. Despite the supply of epitheliotrophic growth factors, the healing time was seldom considered as an outcome in the studies.

The metrics used to score all the parameters were inconsistent throughout the studies, making it not possible to make a meta-analytic comparison.

## 9. Conclusions

Blood-based eye drops represent an emerging treatment option of different ocular surface disorders, as they supply a combination of active substances GFs and cytokines that mimic the function of natural tears.

Allogeneic sources seem to represent a rising innovation, allowing to obtain a customized eye drop on the base of the specific subtype of patients and ocular surface diseases.

However, further randomized clinical trials and internationally recognized harmonized guidelines are still needed to provide better evidence, to improve quality of the final products, and to lead to a more widespread use of these therapies in daily ophthalmological practice. Most importantly, future studies need to include short-, medium-, and long-term follow-up to determine if any benefit is maintained after treatment and for how long.

There remains many unanswered questions regarding what might be considered optimal treatment, sketched as “The 5 Ws (and 2 Hs) for blood-based eye drops”, as follows.

Who is the patient to be treated, in terms of disease type, severity, and stage?

Why is a blood-based treatment needed, in terms of a target indication?

When is it appropriate to prescribe blood-based therapy, as too late is not always a good option?

Where are the products dispensed? Is a national/regional program a feasible solution to optimize resources?

What is the product of choice? Which source and preparation are targeted for a given patient? Is a patient self-report enough, or should the clinician who prescribed the product report the course, as surgeons do in organ transplants?

How is the product standardized in terms of processing to ensure optimal dilution, solvent, dispenser, and storage time?

How is treatment delivered to the ocular surface, in terms of posology, dose-size modulation, length of treatment, and number of cycles?

Preclinical studies are needed to standardize and compare blood based products, and an interdisciplinary work with transfusional medicine specialists is imperative to share information and competences.

## Figures and Tables

**Table 1:** Summary of studies related to Auto-SED. Legend: Auto-SED = Autologous serum eye drops; CIC = Conjunctival impression cytology; CMC = Carboxymethylcellulose; d = day; DED = Dry eye disease; FS = Fluorescein staining; GVHD = Graft versus host disease; I = interventional; ISS = Isotonic Saline Solution; LASIK = Laser assisted in situ keratomileusis; M = Month; NaHy = Sodium hyalutonate; NK = Neurotrophic keratitis; OSDI = Ocular Surface Disease Index; P = Prospective; PED = Persistent epithelial defects; PK = Penetrating Keratoplasty; RCT = randomized controlled trial; RBS = Rose Bengal staining; SS = Sjogren’s syndrome; LSCD = Limbal stem cell deficiency; ST = Schirmer test; TBUT = Tear Break Up Time; wks = weeks.

Study First Name, Year	Evidence Level	Design	Condition	Inclusion Criteria	Patients (*n*)	Controls (*n*)	Control arm	Solvent	Dilution	Posology	Duration of Treatment	Main Outcomes	Results
Tananuvat 2001 [49]	2	RCT	DED	Refractory to treatment (tear substitute, punctum plug)	12	Fellow eye	Saline solution	ISS	20%	6×/d	2 Ms	Subjective symptoms, FS, RBS, and CIC	No significant improvement of symptoms and signs between the two groups
Ogawa 2003 [55]	2	P, I	DED (GVHD)	Refractory to treatment (tear substitute)	14	/	/	ISS	20%	10×/d	4–41 Ms	Subjective symptoms, FS, RBS, TBUT, and Schirmer test	Improvement of symptoms, TBUT, RBS, and FS
Matsumoto 2004 [59]	3	Retrospective, non-comparative case series	NK	Refractory to treatment (therapeutic contact lenses, tear substitute, and sodium hyaluronate)	11	/	/	ISS	20%	5–10×/d	Up to 36 Ms	Changes in corneal disease state, corneal sensitivity, and BCVA	100% healing; 64% increase of corneal sensitivity
Noble 2004 [50]	1	RCT, Cross over	DED	Epitheliopathy with corneal/conjunctival RBS, ST < 5 mm/5 min and punctal occlusion	8 + 8	8 + 8	Conventional treatment	ISS	50%	As previous therapy	3 Ms + 3 Ms	Subjective symptoms, TBUT, ST, FS, clearance test, and CIC	Improvement of symptoms and CIC after Allo-SED
Kojima 2005 [51]	1	RCT	DED	DED symptoms, positive FS or RBS, ST < 5 mm/5 min or TBUT < 5 s	10	10	Preservative-free tear substitute	ISS	20%	6×/d	2 wks	Subjective symptoms, FS, RBS, TBUT, and ST	Improvement of symptoms, TBUT, RBS, and FS in Allo-SED group
Noda-Tsuruya 2006 [60]	1	RCT	Post-LASIK	Patients who received LASIK	12	15	Tear substitute	ISS	20%	5×/d	1 week–6 M	Subjective symptoms, FS, RBS, TBUT, and ST	Improvement of TBUT, FS, and RBS. No change in symptoms
Schulze 2006 [61]	2	RCT	Corneal Epithelial Abrasions in Diabetic Patients	Diabetic patients undergoing pars plana vitrectomy who received corneal abrasion for better intraoperative visualization	13	10	NaHy	ISS	50%	Every hour	Until epithelial healing	Size of abrasion	Faster closure of corneal epithelial wounds
López-Garcia 2008 [66]	2	P, I	LSCD	Aniridic keratopathy	13	/	/	Isotonic saline solution	20%	8×/d	2 Ms	CIC, TBUT, ST, and tear meniscus level	Improvement of symptoms epithelialization, and ST
Ziakas 2010 [62]	2	P, I	Recurrent Corneal Erosion	Refractory to treatment and history of at least three relapses	33	/	/	ISS	20%	6×/d for 3 Ms, then 3×/d for 3 Ms	6 Ms	Subjective symptomsand recurrence	85% healing with no recurrences over the whole follow-up period
Chen 2010 [65]	1	P, I	Post PK	Patients who received PK	82	83	Tear substitute	Tear substitute	20%	8×/d	3 d–2 wks	Corneal epithelial healing	Improvement of healing time
Urzua 2012 [52]	1	RCT, Cross over	DED (NSS)	DED symptoms score more than 40 (OSDI questionnaire), TBUT < 5 s, positive FS or ST < 5 mm/5 min	6 + 6	6 + 6	Tear substitute	ISS	20%	4×/d	2 wks + 2 wks	Subjective symptoms	Improvement of OSDI after Allo-SED
Lekhanont 2013 [63]	2	P, I	PED after ocular surgery	Refractory to treatment	181	/	/	Tear substitute	100%	Every 2 h	Until full healing	Rate of full healing corneal epithelial defect	93% healing within 4 d
Cho 2013 [37]	2	P, I	DED (SS and NSS) PED	DED: Symptoms, TBUT ≤ 5 s, ST ≤ 5 mm/5 min, positive corneal FS PED: Refractory to treatment (tear substitute, patching, and therapeutic contact lenses)	22	Group 2: 35 Group 3: 28	/	ISS; NaHy; Ceftazidime	100% vs. 50%	6×/d	3 Ms	Subjective symptoms, TBUT, ST, FS, and rate of complete healing of PED	In SS and PED, Allo-SED 100% was the most effective in decreasing symptoms and FS and in accelerating healing. In NSS, Allo-SED 100% and 50% were similar in reducing symptoms and FS
Celebi 2014 [53]	1	RCT, Cross-over trial	DED	Refractory to treatment	10 + 10	10 + 10	Preservative-free tear substitute	ISS	20%	4×/d	1 M + 1 M	Subjective sympthoms	Significant improvement of OSDI and TBUT after Allo-SED. No change of corneal damage and ST
Hussain 2014 [38]	2	Retrospective, cohort study	DED	Refractory to treatments (lubrication, topical corticosteroids, cyclosporine 0.05%, and/or punctal occlusion)	63	/	/	ISS	50%	4×/d	3 Ms–48 Ms	Subjective symptoms, FS, TBUT, and ST	Significant improvement of TBUT, FS, and symptoms
Lopez-Garcia 2014 [39]	1	RCT	DED (SS)	Diagnosis of SS	13 + 13	/	/	ISS	20%	3×/d	2 Ms	TBUT, ST, FS, RBS, and CIC	Improvement of symptoms, epithelialization, BUT, and ST in both groups
Hwang 2014 [56]	2	P, I, cross-sectional	DED (SS)	ST < 5 mm/5 min, corneal Staining above 2 (Oxford Scale)	34	/	/	NaHy	50%	8×/d	4 wks	Subjective symptoms, FS, and TBUT	Patients with primary SS had improvements in ocular symptoms, FS, and TBUT. Patients with secondary SS had no improvement.
Jirsova 2014 [58]	2	P, I	DED	ST I < 5 mm/5 min, TBUT < 5 s	17	/	/	ISS	20%	Open, up to 12×/d	3 Ms	Subjective symptoms, FS, CIC, and ST	Significant improvement of ST and symptoms
Semeraro 2016 [57]	2	P, I, case-control	DED (SS)	DED symptoms, ST < 5 mm/min or TBUT < 10 s	12	12	Tear substitute	ISS	50%	5×/d	12 Ms	Tear production, tear stability, and FS	Significant improvement of symptoms
Yilmatz 2017 [54]	1	RCT, cross-over trial	DED (due to isotretinoin)	DED symptoms, TBUT < 10 s, ST < 10 mm/min	24	24	Preservative-free tear substitute	ISS	20%	Not specified	1 M + 1 M	Subjective symptoms, ST, and TBUT	Significant improvement of symptoms and TBUT in Allo-SED group
Sul 2018 [64]	1	P, I, case-control	Post Pterygium excision	Patients who received Pterygium Excision	25	25	Tear substitute	CMC	50%	8×/d	3–8 d	Subjective symptoms and corneal epithelial healing	Improvement of healing time and symptoms

**Table 2:** Summary of studies related to Allo-SED from adult donors. Legend: Allo-SED = Allogenic serum eye drops; CMC = Carboxymethylcellulose; d = day; DED = Dry eye disease; FS = Fluorescein staining; GVHD = Graft versus host disease; I = interventional; ISS = Isotonic Saline Solution; OSDI = Ocular Surface Disease Index; P = Prospective; PED = Persistent epithelial defects; ST = Schirmer test; TBUT = Tear Break Up Time; wks = weeks.

Study First Name, Year	Evidence Level	Design	Condition	Inclusion Criteria	Patients (*n*)	Controls (*n*)	Control Arm	Solvent	Dilution	Posology	Duration of Treatment	Main Outcomes	Results
Chiang 2009 [69]	2	P,I	PED	PED since >2 wks refractory to treatment (tear substitute and soft contact lenses)	36	/	/	/	100%	Every hour	Variable	Healing	Complete healing in 42% within 2 weeks
Na and Kim 2012 [72]	2	P,I	DED (GVHD)	Refractory to treatment	16	/	/	CMC and ofloxacin	20%	6–8×/d	4 wks	Subjective symptom, TBUT, ST, FS, tear osmolarity, corneal staining, and CIC	Improvement in OSDI, FS, IC, and tear osmolarity
Harritshøj 2014 [71]	3	Retrospective cohort	DED, PED	Refractory to treatment	DED 20 PED 14	/	/	ISS	20%	6×/d	2–4 wks	Subjective symptoms and healing	Improvement of symptoms and FS in DED group at 4 wks. No Improvement in PED group

**Table 3:** Summary of the studies on the use of UCBS. Legend: Auto-SED = Autologous serum eye drops; AMT = Amniotic membrane transplantation; BSS: Balanced salt solution; CIC = Conjunctival impression cytology; CMC = Carboxymethylcellulose; d = day; DALK = Deep anterior lamellar keratoplasty; DED = Dry eye disease; FS = Fluorescein staining; GVHD = Graft versus host disease; I = interventional; ISS = Isotonic Saline Solution; LASEK = laser epithelial keratomileusis; M = Month; OSDI = Ocular Surface Disease Index; P = Prospective; PED = Persistent epithelial defects; PK = Penetrating Keratoplasty; RCT = randomized controlled trial; RBS = Rose Bengal staining; SS = Sjogren’s syndrome; ST = Schirmer test; TBUT = Tear Break Up Time; wks = weeks.

Study First Name, Year	Evidence Level	Design	Condition	Inclusion Criteria	Patients (*n*)	Controls (*n*)	Control Arm	Solvent Composition	Dilution	Posology	Duration of Treatment	Main Outcomes	Results
Vajpayee 2003 [88]	1	RCT	PED	Refractory to treatments (tear substitute, patching, and contact lens)	31	29	Auto-SED	ISS	20%	6×/d	3 wks	BCVA, FS, TBUT, ST, and PED size	Improvement of re-epithelization rate in UCBS group
Yoon 2005 [89]	3	P, I	PED	PED refractory to treatments; persisted for at least 2 weeks	14	/	/	ISS	20%	6×/d	2 wks–1 M	PED area	43% healing within 2 weeks; 43% healing within 4 weeks
Yoon 2006 [82]	2	P, I	DED	Refractory to treatments. DED symptoms > 3 months, TBUT < 5 s, ST < 5 mm/min, positive FS or RBS	31	/	/	ISS	20%	6–10×/d	2 Ms	Subjective symptoms, TBUT, ST, corneal sensitivity, and FS	Improvement of symptoms, TBUT, and FS
Yoon 2007 [90]	2	P, I	DED	Refractory to treatments. DED symptoms > 3 months, TBUT < 5 s, ST < 5 mm/min, positive FS or RBS	27	21	Auto-SED	ISS	20%	6–10×/d	2 Ms	Symptom scoring, corneal sensitivity, TBUT, ST, tear clearance rate, FS, and CIC	Symptoms, TBUT, FS, and CIC findings improved in both groups. Symptoms and FS were lower in the CBS group
Yoon 2007 [93]	2	P, I	DED (GVHD)	Refractory to treatments, TBUT < 5, ST < 5 mm/min, positive FS	12	/	/	ISS	20%	6–10×/d	6 Ms	Subjective symptoms, TBUT, ST, corneal sensitivity test, FS, and tear clearance rate	Significant improvement in symptoms, corneal sensitivity, TBUT, and FS
Yoon 2007 [98]	2	P, non-comparative case series	NK	Refractory to treatment (tear substitute and contact lens)	28	/	/	ISS	20%	6×/d	2–4 wks	Epithelial healing time, BCVA, and corneal sensitivity	100% corneal healing after 4 weeks
Sharma 2011 [91]	1	RCT	Acute Chemical Burn	Acute chemical burns of grades III, IV, and V (Dua’s classification)	Group 1: 12	Group 2: 11 Group 3: 10	Group 2: Auto-SED Group 3: Medical Treatment	ISS	20%	10×/d	3 Ms	Time to epithelialization, subjective symptoms, PED area, extent of limbal ischemia, corneal clarity, and symblepharon formation	Significant reduction of time to epithelialization after CBS therapy compared to AS and medical treatment
Yoon 2011 [94]	2	P, I, case-control	RCE	History of RCE	18	17	Tear substitute	ISS	20%	4–6×/d	1 year	Number of recurrences	Reduction of recurrences in UCBS group
Versura 2013 [46]	2	P, I	PED (GVHD and SS)	DED symptoms, positive FS or RBS, ST < 5 mm/5 min or TBUT < 5 s	30	/	/	Phosphate buffered saline	20%	8×/d	1 M	PED area, subjective symptom (OSDI), ST I, TBUT, tear osmolarity, corneal esthesiometry, and CIC	Significant reduction of epithelial damage
Yoon 2013 [96]	2	P, I, case-control	Epithelial defect post-LASEK	Patients underwent LASEK	32	28	Tear substitute	ISS	20%	4–6×/d	3 Ms	Epithelial healing time, BCVA, Haze score (0–4), ST, and TBUT	Improvement of corneal haze and tear film parameters
Erdem 2014 [87]	2	P, I	PED	Refractory to treatment (tear substitute and patching contact lens)	14	/	/	ISS	20%	10×/d then 5x/d	3 wks	PED area	75% healing within 12 ds
Mukhopadhyay 2015 [97]	1	RCT	DED (Hansen’s disease)	ST < 5 mm/5 min	48	Group B: 52 Group C: 44	Group B: Auto-SED 20% Group C: Tear substitute	ISS	20%	6–10×/d	6 wks	Subjective symptom, ST I, TBUT, and CIC	Better improvement of clinical parameters in CBS group
Sharma 2016 [92]	1	RCT	Acute Chemical Burn	Acute chemical burns of grades III, IV, and V (Dua’s classification)	Group 3: 15	Group 1: 15 Group 2: 15	Group 1: Medical treatment Group 2: AMT, Medical treatment	/	20%	10×/d	Open	Subjective symptoms, TBUT, and ST	UCS and AMT are equally efficacious
Giannaccare 2017 [86]	2	P, I	DED	DED symptoms, positive FS or RBS, ST < 5 mm/5 min or TBUT < 5 s	20	/	/	ISS	20%	8×/d	4 Ms	Subjective symptom (OSDI), ST I, TBUT, FS, and corneal sensitivity	Significant improvement of all clinical parameters
Kamble 2017 [95]	1	RCT	Epithelial defect post-keratoplasty	Epithelial defect post PK and DALK	Group 1: 35	Group 2: 35 Group 3: 35	Group 2: Auto-SED 20% Group 3: Tear substitute	BSS	20%	6×/d	Until healing	Rate of re-epithelialization	Rate of re-epithelialization comparable between CBS and Auto-SED groups

**Table 4:** Summary of the studies on the use of preparations from PRP (Platelet-Rich Plasma); PL = Platelet Lysate; PRGF = Platelet Rich in Growth Factors. Legend: Allo-SED = Allogenic serum eye drop; AMT = Amniotic membrane transplantation; CIC = Conjunctival impression cytology; d = day; DED = Dry eye disease; GVHD = Graft versus host disease; I = interventional; ISS = Isotonic Saline Solution; LASIK = Laser assisted in situ keratomileusis; M = Month; NaHy = Sodium hyalutonate; NK = Neurotrophic keratitis; OSDI = Ocular Surface Disease Index; P = Prospective; PED = Persistent epithelial defects; PRGF = plasma rich in growth factors; PRP = Platelet rich plasma; PL = Platelet lysate; RCT = Randomized controlled trial; RBS = Rose Bengal staining; SS = Sjogren’s syndrome; ST = Schirmer test; TBUT = Tear Break Up Time; wks = weeks.

Study, First Name, Year, [ref]	Evidence Level	Product	Design	Disease	Inclusion Criteria	Patients (*n*)	Controls (*n*)	Control Arm	Solvent	Dilution	Posology	Duration of Treatment	Main Outcomes	Results
Kim 2012 [109]	2	PRP	Retrospective	PED	Refractory to treatment (Tear substitute)	28	17	Allo-SED	ISS	20%	4×/d	Until healing	Epithelial healing time and rate	Higher healing rate in PRP group
Alio 2007 [104]	2	PRP	P, I	DED	FS > 50%, clinical signs of inflammation	18	/	/	ISS	/	4–6×/d	1 M	Subjective symptoms, BCVA, tear meniscus height, TBUT, FS, and CIC	Improvement of symptoms (89%), BCVA (28%), tear meniscus height (56%), TBUT (50%), and FS (72%)
Alio 2007 [106]	2	PRP vs. AMT	P, I	Corneal ulcers	Refractory to treatment	38	/	/	ISS	/	6×/d	6 Ms	Ulcer size, inflammation, healing, BCVA, and subjective symptoms	Improvement of signs and symptoms
Alio 2007 [105]	3	PRP	P, I	DED post-LASIK	Patients who received LASIK	13	/	/	ISS	/	6×/d	1 M	Subjective symptoms, BCVA, FS, and TBUT	Improvement of symptoms (85%); BCVA from 1 to 2 lines (54%); disappearance of FS (69%); increase of TBUT > 2 s (46%)
Lee 2016 [112]	2	PRP	Retrospective	RCE	Patients treated with conventional therapy	47	20	Tear substitute	ISS	20%	Every 2 h for 2 Ms, 4×/d for 4 Ms	6 M	Recurrence rate	Reduced recurrence rate in PRP group
Panda 2012 [110]	1	PRP	RCT	Chemical injury	/	20	10	Tear substitute	ISS	/	10×/d	3 Ms	Corneal transparency and BCVA	Significant improvement of corneal transparency and BCVA in PRP group
García-Conca 2018 [118]	1	PRP	RCT	Hyposecretory DED	ST < 5.5 mm, OSDI ≥ 13, Oxford scale score ≥ 1	83	39	NaHy, Tear substitute	ISS	/	6×/d	30 ds	ST, tear osmolarity, FS, TBUT, conjunctival hyperaemia, OSDI, and CIC	Improvement of signs in PRP group
Avila 2018 [120]	1	PRP injected	RCT	DED (SS)	Patients who did not receive medications like ciclosporin or topical steroids or lacrimal plugs and lacrimal occlusion	30	15	Tear substitute	ISS	/	1 mL PRP		FS and TBUT	Improvement of lacrimal production and TBUT; reduction FS in PRP group
Wróbel-Dudzińska 2018 [117]	2	PRP	P, I	NK	Refractory to treatment	25	/	/	/	/	5×/d	3 Ms	BCVA, healing of corneal surface, subjective symptoms, and corneal thickness	Improvement of BCVA; PED full healing (80%); lack of discomfort and photophobia (96%); no progression of corneal damage
Pezzotta 2017 [102]	2	PL	P	DED (GVHD)	Refractory to treatment (tear substitute for at least 3 Ms)	23	/	/	/	/	4×/d	6 Ms	Symptoms, TBUT, and FS	Improvement of symptoms (74%), TBUT (86.9%), and FS (69.6%)
Fea 2016 [111]	2	PL	P, case-control	DED (SS)	Severe DED, OSDI ≥ 23, Oxford scale score ≥ 1; refractory to treatment for more than 2 Ms (tear substitute, steroids, cyclosporine A, or allo-SED)	30	10	Tear substitute	ISS	50%	4×/d	3 Ms	OSDI, ST, FS, BCVA, and TBUT	Improvement of OSDI, FS, and TBUT in PL group
Zallio 2016 [103]	2	PL	P, I	DED (GVHD)	Recent diagnosis of GVHD	26	/	/	BSS	30%	6×/d	1 year	/	Improvement in symptoms (91%); remission of corneal damage, (86%) and improved National Institutes of Health scores (73%)
Lopez-Plandolit 2010 [107]	2	PRGF	P, I	PED	Refractory to medical and surgical treatments	18	/	/	ISS	/	Every 2 h for 3 d, then variable	Until healing	Epithelial healing rate and time	85% healing within a mean of 11 weeks
Sanchez-Avila 2017 [101]	2	PRGF	Retrospective	DED (SS)	SS	26	/	/	ISS	/	4×/d	6 wks (= 1 cycle)	OSDI, VAS, and BCVA	Improvement of OSDI score, BCVA, VAS frequency, and VAS severity
Sanchez-Avila 2018 [114]	2	PRGF	Retrospective	NK stage 2 and 3	ST < 5 mm, TBUT < 5 s, severity of subjective symptoms in the level of severity of dry eye	31	/	/	ISS	/	4×/d	6 wks (1 cycle)	Ulcer closure at 4 weeks, OSDI, VAS, and BCVA	Resolution of corneal defect/ulcer (97.4%) in 11.4 weeks; reduction of OSDI (60.9%), VAS frequency (59.9%), and VAS severity (57%); improvement of BCVA (52.8%)
Sanchez-Avila 2018 [115]	2	PRGF	retrospective, comparative, and descriptive	DED post-LASIK	Patients who received LASIK	79	39	Tear substitute	ISS	/	4×/d	6 wks (1 cycle)	VAS, OSDI, BCVA, TBUT, ST, and IOP	Improvement in OSDI (38.12%), VAS (41.89%), severity (42.47%), and ST (88.98%) in PRGF group
Sanche-Avila 2018 [116]	2	PRGF	Retrospective	DED (GVHD)	Refractory to treatments for 3 Ms (tear substitute, topical/oral antibiotics, corticoids and antivirals, contact lens, punctal occlusion, Allo-SED, cyclosporine, and AMT)	12	/	/	ISS	/	4×/d	6 wks (1 cycle)	Resolution of corneal ulcers	Improvement in the area (75.7%) and density (73.3%) corneal staining, BCVA (74.7%), OSDI (75.4%), visual analog score frequency (81.4%), and VAS severity (81.9%), and an increase of 3.8 s in TBUT and 6 mm in ST
Merayo-Lloves 2016 [113]	2	PRGF	Retrospective	Evaporative DED	Refractory to treatment (tear substitute, topical or/and systemic corticosteroids, AS, or cyclosporine)	83	/	/	ISS	/	4×/d	6 wks	OSDI, BCVA, VAS, and ST	Reductions in the OSDI (38.2%), BCVA (27.4%), and VAS for frequency (32%) and severity (34%) and improvement in ST (177.5%)
